# LS-NMF: A modified non-negative matrix factorization algorithm utilizing uncertainty estimates

**DOI:** 10.1186/1471-2105-7-175

**Published:** 2006-03-28

**Authors:** Guoli Wang, Andrew V Kossenkov, Michael F Ochs

**Affiliations:** 1 Division of Population Science, Fox Chase Cancer Center, Philadelphia, PA, USA

## Abstract

**Background:**

Non-negative matrix factorisation (NMF), a machine learning algorithm, has been applied to the analysis of microarray data. A key feature of NMF is the ability to identify patterns that together explain the data as a linear combination of expression signatures. Microarray data generally includes individual estimates of uncertainty for each gene in each condition, however NMF does not exploit this information. Previous work has shown that such uncertainties can be extremely valuable for pattern recognition.

**Results:**

We have created a new algorithm, least squares non-negative matrix factorization, LS-NMF, which integrates uncertainty measurements of gene expression data into NMF updating rules. While the LS-NMF algorithm maintains the advantages of original NMF algorithm, such as easy implementation and a guaranteed locally optimal solution, the performance in terms of linking functionally related genes has been improved. LS-NMF exceeds NMF significantly in terms of identifying functionally related genes as determined from annotations in the MIPS database.

**Conclusion:**

Uncertainty measurements on gene expression data provide valuable information for data analysis, and use of this information in the LS-NMF algorithm significantly improves the power of the NMF technique.

## Background

Because of their ability to link genes that behave similarly across conditions in a gene expression study, pattern recognition and clustering are widely used for data analysis of microarray data (see [1] for a review). Numerous methods have been adopted to cluster genes or samples including hierarchical clustering [2], maximum likelihood clustering [3], the cluster affinity search technique [4], quality threshold clustering [5], and fuzzy K-means clustering [6], among others. Some methods specifically aim to identify subsets of behaviors within the data, where genes behave similarly only over a subset of samples. Such methods include two-way clustering [7] and biclustering [8]. Other methods allow genes to belong to multiple patterns, reflecting biological roles where genes function in multiple cellular processes. Such methods include Bayesian Decomposition [9,10], principal component analysis [11], independent component analysis [12], and nonnegative matrix factorization [13-15].

By creation of a constrained model (e.g., a model with only positive points), non-negative matrix factorization (NMF) shares with Bayesian Decomposition (BD) the potential to more accurately identify sets of genes that together provide function. Both aim to recover two matrices, **A **and **P**, that reproduce the data within the uncertainty as in

**D **= **M **+ *ε *= **A**·**P **+ *ε*.     (1)

In the case of NMF, this constraint is positivity in the **A **and **P **matrices, while in BD the constraint is provided through relationships between model points in the form of convolution functions during matrix reconstruction [16]. For microarray data, the matrix **D **provides the estimates of transcriptional levels, such that each column corresponds to the estimate for a single condition, with each matrix element in a column corresponding to the estimate for a single gene (or probe set) in that condition. A row of **D **corresponds to the processed intensity for a single gene across all conditions. If **D **has dimension of *I *× *J*, then **A **has dimensions *I *× *K*, and **P **has dimensions *K *× *J*, where *K *is the dimensionality (i.e., rank). There is no accepted method yet to choose *K a priori*, however many possible *K *can be tested in order to find an optimal value [17].

The overall simulation in the original NMF algorithm aims to minimize the difference between **M **and **D **in Equation 1, with every element in **D **given the same weight in evaluating the difference between the two matrices. However, most microarray measurements are replicated and, in addition, statistical techniques have been developed to estimate uncertainty measurements for all data points in **D **[18-21]. This uncertainty information is extremely valuable for identifying the best model [10], and it has been used in microarray analysis in other contexts as well [22]. Inclusion of this information in fitting the **A **and **P **matrices should improve the NMF algorithm by allowing it to more precisely fit the reconstructed **M **to the data, **D**. Such approaches have also been used successfully within supervised methods, such as LS-SVM [23].

## Results

### Data sets

We applied the least squares nonnegative matrix factorization (LS-NMF) algorithm to two publicly available microarray datasets, one with no individual estimates of uncertainties for each data point and one with such information. The first set is yeast cell cycle data from cultures synchronized with a temperature sensitive cdc28-mutant [24], which has a single Affymetrix GeneChip measurement at each time point. The second set is the yeast deletion mutant compendium from Rosetta Inpharmatics [25], which comprises microarray measurements of mRNA levels from yeast cultures containing either clones of *S. cerevisiae *with gene deletions or chemical treatments. Both data sets were preprocessed as described in Methods to create the estimates of mRNA levels for the data matrix, **D**.

### Algorithm performance

#### Data fitting

**Figure 1:**
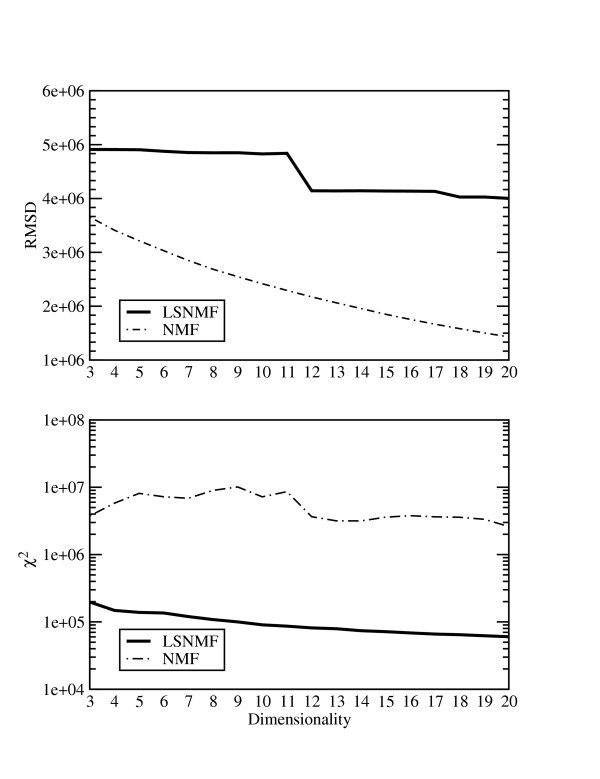
**Fits to the Data**. The comparison of LS-NMF and NMF for fitting data is shown for the Rosetta Compendium. NMF is based on root mean squared deviation (RMSD) fitting, and as the number of dimensions increases, the fit improves with potential overfitting possible. The *χ*^2 ^measurement provides the standard measure of fit to the data and shows how the LS-NMF method fits data much better once individual uncertainty measurements are considered.

**Figure 2:**
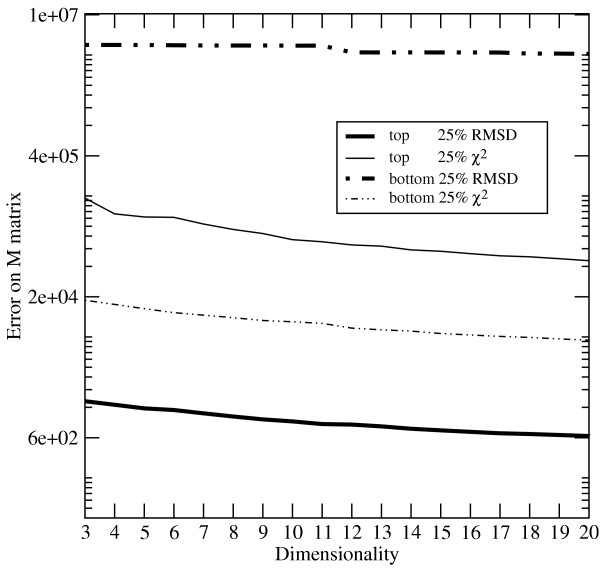
**Misfit in M**. The error between portions of the **M **and **D **matrices is shown as the dimensionality increases for the Rosetta data as fit by LS-NMF. The bottom 25% of the genes in terms of precision (i.e., highest variance) are shown together with the top 25%. Since LS-NMF relies on the variance, the algorithm fits the reliable data with improving accuracy, while ignoring poor data in terms of RMSD as the dimensionality increases.

To measure the ability of LS-NMF to fit the data, we measured the *χ*^2 ^fit (as in Equation 6) between the model of the data provided by **M **and the data in **D**, as in Equation 1. The original NMF algorithm used the root mean square distance (RMSD) between these two matrices as a measure of fit, however this is incapable of taking into account the uncertainty information used by LS-NMF. Essentially, NMF ignores variations in the precision of the measurements. The *χ*^2 ^measure provides a standard approach to determining the fit between a model of a data set and the data set itself. Figure 1 shows the difference between the **D **and **M **matrices using LS-NMF and NMF for dimensionalities of *K *= 3 - 20 on the Rosetta data set. Figure 1 demonstrates that the *χ*^2 ^error value decreased with increasing dimensionality for LS-NMF, but not for the NMF simulation. At all dimensions, LS-NMF consistently fits the data better, which is expected as NMF does not optimize **M **for the known variance. More interestingly, in the LS-NMF simulation, the RMSD error appears to be independent of the dimensionality, while NMF does depend on the dimensionality. This results from the LS-NMF simulation fitting the more reliable data points tightly, while allowing the data points with high variance to be fit more loosely. This can be seen in Figure 2, where points with the highest variance (bottom 25%) do not improve in RMSD in LS-NMF, while they continue to show improvement in *χ*^2^. The *χ*^2 ^measure takes into account the variance providing a better measure of fit in cases of varying variance.

#### Computational complexity

While incorporating uncertainty measurements into NMF update rules (as described in Methods) makes sense for fitting microarray data, if the modification adds too much computational complexity, it will not be useful practically. We have measured the additional computational cost when applied to the Rosetta data set, and we have found that it is a constant offset compared to the cost of the original NMF implementation, regardless of complexity. This indicates that LS-NMF has the same convergence speed as NMF after a higher set-up cost, so that LS-NMF can be used with a minimal loss of efficiency compared with the original NMF algorithm in most practical applications.

### Biological insights

#### ROC analysis of metagenes

After normalization as described in the Methods, the contribution of each gene to a metagene is represented by a scaled *Z*-score, with a positive *Z*-score indicating that the gene is likely to be associated with that metagene, and a negative *Z*-score indicating that the gene is unlikely to be associated with the metagene. Metagenes were introduced to summarize behavior shared by many genes within an experiment that together provided the ability to classify samples [26,27]. This was similar to the concept of an eigengene from singular value decomposition [11], but with regression models using classification data driving the determination of mixing of the genes in a metagene. Metagenes have been discussed in relation to NMF analysis of microarray data previously [15], where they summarize behavior across conditions and assign fractions of the overall expression pattern for each gene to these behaviors.

**Figure 3:**
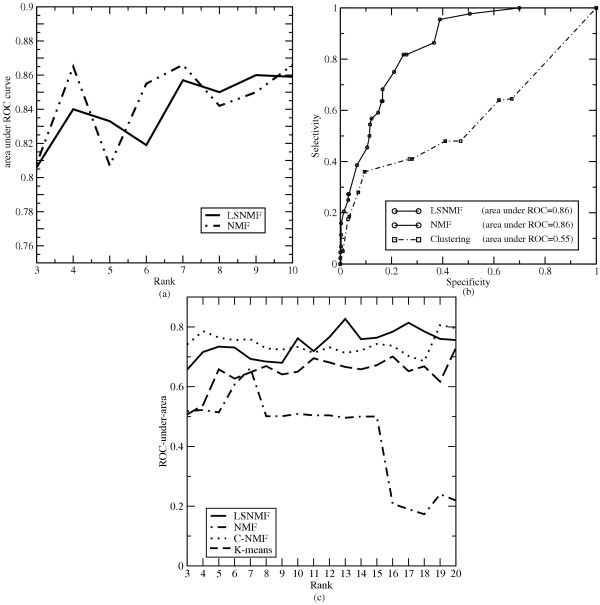
**ROC Analysis of Yeast Cell Cycle and Rosetta Data Sets**. ROC analysis for NMF and LS-NMF for the yeast cell cycle data is summarized in sections a and b, while section c shows the results for the Rosetta Compendium analysis, where variance estimates are available. In a, the total area under the ROC curves is shown for LS-NMF (solid line) and NMF (dashed line). In b, LS-NMF and NMF are compared to hierarchical clustering at a single dimensionality (the LS-NMF and NMF curves are superimposed as there is no difference). In c, the areas under the ROC curve for analysis of the Rosetta data are shown for LS-NMF (solid line), NMF (dash-dot line), K-means clustering (dashed line), and NMF on scaled data (C-NMF, dotted line). Here the variance information allows far better results to be obtained, either by scaling or by use of LS-NMF. The advantages of the approach used in LS-NMF are discussed in the text.

Figure 3 shows that metagenes can recover known gene coregulation in the well studied yeast cell cycle data set [24]. The ROC test for the cell cycle data set relies on known sets of coregulated genes [28] and is described in Methods. Figure 3a shows the areas under the ROC curve against the number of metagenes (dimensions) for both NMF and LS-NMF. Because the cell cycle data set does not have uncertainty estimates, we use a uniform multiplicative uncertainty estimate in LS-NMF (see Equations 6–8). As expected, LS-NMF does not outperform NMF here, as there is no uncertainty information. The differences between the curves merely reflects random differences in changes generated by slightly different update rules. To verify that LS-NMF behaves like NMF in the limit, we assigned unit uncertainty to all data points, which should reduce LS-NMF to NMF, as Equations 7–8 reduce to Equations 2–3. We analyzed the cell cycle data using six dimensions in agreement with our earlier work [9] and compared the performance of LS-NMF and NMF to shrinkage-based hierarchical clustering [28]. As can be seen in Figure 3b, both NMF and LS-NMF clearly perform much better at the recovery of known biological coregulation groups (note that the curves for NMF and LS-NMF lie on top of each other). We used the Rosetta compendium [25] to explore the ability of LS-NMF to gain power from uncertainty data, with the gold standard supplied by genes that together provide a metabolic pathway (see Table 1). Figure 3c shows the performance of NMF and LS-NMF across different values of dimensionality *K *in terms of area under the ROC curve, as well as results for K-means clustering applied to scaled data and for NMF applied to scaled data. The scaled data set was generated by dividing each data point in **D **by its associated uncertainty as in the original publication [25]. For all dimensionalities, LS-NMF improves the recovery of coregulated genes by 15% over NMF and K-means. Scaling the data has an effect similar to LS-NMF (see update equations below), however this leads to problems in interpretation of the metagenes, since the amplitude of a gene in a metagene will be reduced to near zero for a gene with strongly scaled data (see Equation 1).

#### Prediction of functional relationships

The other way to evaluate the performance of NMF and LS-NMF is by testing their ability to predict functional relationships between genes. In the reduced Rosetta data set, 215 conditions are measurements of mRNA levels in deletion mutants of *S. cerevisiae *growing in rich media compared with mRNA levels of wildtype *S. cerevisiae *in similar conditions. Mutants showing similar changes in gene expression might be expected to have deletions of functionally related genes, allowing predictions of functional relationships between genes based on links between deletion mutants. These predictions were scored against available database information. In order to demonstrate the gain from including uncertainty measurements, predictions based on **P **matrices in Equation 1 from NMF, LS-NMF, and NMF on data scaled by the uncertainty estimates were compared at different dimensions. The dimensions chosen match previous work using estimation by Bayesian Decomposition and ClutrFree [17], where 15 dimensions were estimated to cover the data, and NMF [13], where 50 dimensions were estimated. In addition, correlations in the original data space (**D**) were also calculated as a baseline providing estimates of the ability of the data to predict functional relationships independent of any dimensionality reduction.

**Figure 4:**
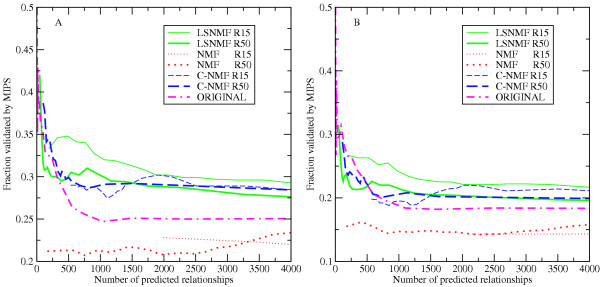
**Comparisons of Metagenes and MIPS Functional Classes**. Evaluation of metagenes for LS-NMF, NMF, and NMF on scaled data, as well as expression profiles for unreduced data, was done by determining the ability of each approach to find functionally related genes based on the MIPS functional classification. In a, the fraction of correct classifications at level 2 in the MIPS ontology are shown in terms of increasing numbers of gene pairs (i.e., decreasing threshold for correlation). In b, the same information is shown for level 3 in the MIPS ontology. The performance of NMF can be improved by utilizing a divergence based update rule, however performance is still significantly below LS-NMF, with recovery ranging from 12 to 30 times greater with LS-NMF.

Predictions of functional relationships were made using pairwise Pearson correlations between experiments measured in each of the seven spaces (the original data space, and the 15 and 50 dimensional spaces with NMF, LS-NMF, and NMF on scaled data). In all spaces, only the 215 deletion mutant conditions were used for analysis. Predictions were checked against the MIPS database (see Methods), and the results are shown in Figure 4. For each of the methods, Figure 4 shows the percentage of predictions validated by MIPS as a function of the number of predictions made, which increases as the threshold for correlation is lowered. In general, the methods should exhibit the highest validation for their strongest predictions (i.e., highest thresholds, far left in Figure 4), but predictions based on the 15-dimensional NMF did not show such a trend, while 50-dimensional NMF and all LS-NMF did behave as expected. NMF applied to the scaled data and LS-NMF performed similarly at 50 dimensions, but LS-NMF performed better at 15 dimensions. Both produced better results than NMF applied to unsealed data and the unreduced original data. The better consistency of LS-NMF across changes in dimension may indicate that including uncertainty information into NMF updating rules improves the robustness of the algorithm. Note that for all methods except NMF applied to unsealed data, the methods perform at roughly the same level when only the most reliable predictions are considered (left side of figures).

## Discussion

In the last several years, many analytical approaches have been used to identify groups of genes related by their similar expression profiles across different conditions, including time series, tumor samples, or different tissues. Since evolution has led to the borrowing of genes for use in multiple biological functions, the ability of NMF to estimate an expression profile as a linear combination of metagenes gains power by matching biological behavior. This power is demonstrated by the analysis of the yeast cell cycle data, where the ROC analysis shows that NMF is more powerful than hierarchical clustering at recovering coexpression groups. Nevertheless, as shown by the 63 replicated controls in the Rosetta compendium, the mRNA levels of individual genes are not equally well controlled in biological systems, leading to potentially large differences in the variance of mRNA levels between different genes. The constantly improving quality of microarrays and the ability to replicate conditions, either through repeated experimentation in model systems or through the capture of multiple related samples, provides estimates of this gene and condition specific variance. By using this valuable information in NMF update rules, the least squares non-negative matrix factorization (LS-NMF) algorithm improves the ability of this approach to recover biological knowledge as demonstrated by the analysis of the Rosetta compendium. Here, unlike in the yeast cell cycle data, individual uncertainty estimates are available at each data point. The value of this information is demonstrated in Figure 3c, where LS-NMF outperformed NMF in ROC analysis, and in Figure 4, where at both dimensionalities LS-NMF greatly increased the number of successfully recovered functional relationships.

LS-NMF may also be more stable than NMF in interpreting biological functions of genes based on the metagenes when the dimensionality is poorly estimated. In Figure 4, analysis at 15 and 50 dimensions give similar results in gene function prediction for LS-NMF, but not for NMF, where analysis at 15 dimensions failed to give meaningful results. This may result from the lack of uncertainty information in NMF, which makes each data point equally important in feedback to the update rules. A higher dimensionality could then yield a better factorization, while dimensionalities under some threshold would be highly influenced by the noise from mRNA levels with high variance. In LS-NMF, data points with low vairance will always influence the update rules more strongly.

LS-NMF gains its power from inclusion of uncertainty information. Such information can also be added by scaling the data by the uncertainty estimate, as was done in the original Rosetta study [25]. As can be seen in Figures 3 and 4, this gives similar though not identical results. However, the direct inclusion of uncertainty information provides both improved interpretation (15 dimensions in Figure 4) and more flexibility. The direct use of uncertainty information in LS-NMF allows extensions, such as treatment of individual data points separately based on additional information during updating (e.g., *a priori *biological knowledge linking genes), which scaling cannot include. In addition, the approach allows for straightforward addition of methods such as simulated annealing [29], which may be useful in escaping local maxima in the probability distribution.

## Conclusion

We have implemented a new algorithm, LS-NMF, based on NMF, to analyze microarray data. The incorporation of uncertainty information into the analysis of mRNA transcript levels significantly improves the recovery of biological information in the form of functional links between genes. In cases where there is no variance information available, LS-NMF reduces back to NMF. LS-NMF will provide the community with a powerful new tool for analysis of high-throughput data. The implementation is straightforward, so analysis of new data types with similar variance estimates should be possible, such as mass spectrometry data. The source code and documentation is available from  by following the Open-Source link.

## Methods

### The LS-NMF algorithm

LS-NMF, like NMF, operates on preprocessed data from a set of expression array experiments. The data comprises estimates of mRNA transcript levels (single channel) or ratios (two channel) represented as a single matrix **D**. Each row of **D **contains the mRNA estimates for each gene in all conditions (e.g., distinct tissues, experiments, timepoints), and each column corresponds to the estimates of mRNA levels for all genes in a single condition. For a dataset comprising *I *genes with expression measured in *J *conditions, the dimensionality of matrix **D **would be *I *× *J*. The goal of the NMF simulation is to find a small number of metagenes (the number of metagenes provides a dimensionality estimate), each defined as a positive linear combination of *I *genes. The mRNA level estimates across conditions for each gene can be approximated then as a positive linear combination of these metagenes. Mathematically, this can be expressed as an approximate factorization of matrix **D **into a pair of matrixes **A **and **P **as in Equation 1. The mock data, **M**, is the approximation of **D**, based on our estimates of **A **and **P**. The matrix *ε *provides for the error in the measurements in **D**. For *K *metagenes (i.e., *K *dimensions), matrix **A **is of size *I *× *K *with each of the *K *columns defining a metagene. The value of element *A*_*ik *_indicates how strongly gene *i *is associated with metagene *k*. Matrix **P **is then of size *K *× *J*, with each row representing the relative mRNA levels of a metagene across the conditions. The value of element *P*_*kj *_givens the strength of metagene *k *in condition *j*.

For NMF simulation, random matrices **A **and **P **are initialized according to some scheme. For instance, they could be populated from a uniform distribution *U *[0,1]. The two matrices are then iteratively updated using the rules [13,30],

Pαμ←Pαμ∑iAiαDiμ∑iAiαMiμ     (2)
 MathType@MTEF@5@5@+=feaafiart1ev1aaatCvAUfKttLearuWrP9MDH5MBPbIqV92AaeXatLxBI9gBaebbnrfifHhDYfgasaacH8akY=wiFfYdH8Gipec8Eeeu0xXdbba9frFj0=OqFfea0dXdd9vqai=hGuQ8kuc9pgc9s8qqaq=dirpe0xb9q8qiLsFr0=vr0=vr0dc8meaabaqaciaacaGaaeqabaqabeGadaaakeaacqWGqbaudaWgaaWcbaacciGae8xSdeMae8hVd0gabeaakiabgcziSkabdcfaqnaaBaaaleaacqWFXoqycqWF8oqBaeqaaOWaaSaaaeaadaaeqaqaaiabdgeabnaaBaaaleaacqWGPbqAcqWFXoqyaeqaaOGaemiraq0aaSbaaSqaaiabdMgaPjab=X7aTbqabaaabaGaemyAaKgabeqdcqGHris5aaGcbaWaaabeaeaacqWGbbqqdaWgaaWcbaGaemyAaKMae8xSdegabeaakiabd2eannaaBaaaleaacqWGPbqAcqWF8oqBaeqaaaqaaiabdMgaPbqab0GaeyyeIuoaaaGccaWLjaGaaCzcamaabmaabaGaeGOmaidacaGLOaGaayzkaaaaaa@534E@

Aδα←Aδα∑jDδjPαj∑jMδjPαj     (3)
 MathType@MTEF@5@5@+=feaafiart1ev1aaatCvAUfKttLearuWrP9MDH5MBPbIqV92AaeXatLxBI9gBaebbnrfifHhDYfgasaacH8akY=wiFfYdH8Gipec8Eeeu0xXdbba9frFj0=OqFfea0dXdd9vqai=hGuQ8kuc9pgc9s8qqaq=dirpe0xb9q8qiLsFr0=vr0=vr0dc8meaabaqaciaacaGaaeqabaqabeGadaaakeaacqWGbbqqdaWgaaWcbaacciGae8hTdqMae8xSdegabeaakiabgcziSkabdgeabnaaBaaaleaacqWF0oazcqWFXoqyaeqaaOWaaSaaaeaadaaeqaqaaiabdseaenaaBaaaleaacqWF0oazcqWGQbGAaeqaaOGaemiuaa1aaSbaaSqaaiab=f7aHjabdQgaQbqabaaabaGaemOAaOgabeqdcqGHris5aaGcbaWaaabeaeaacqWGnbqtdaWgaaWcbaGae8hTdqMaemOAaOgabeaakiabdcfaqnaaBaaaleaacqWFXoqycqWGQbGAaeqaaaqaaiabdQgaQbqab0GaeyyeIuoaaaGccaWLjaGaaCzcamaabmaabaGaeG4mamdacaGLOaGaayzkaaaaaa@5318@

Mij=∑kAikPkj     (4)
 MathType@MTEF@5@5@+=feaafiart1ev1aaatCvAUfKttLearuWrP9MDH5MBPbIqV92AaeXatLxBI9gBaebbnrfifHhDYfgasaacH8akY=wiFfYdH8Gipec8Eeeu0xXdbba9frFj0=OqFfea0dXdd9vqai=hGuQ8kuc9pgc9s8qqaq=dirpe0xb9q8qiLsFr0=vr0=vr0dc8meaabaqaciaacaGaaeqabaqabeGadaaakeaacqWGnbqtdaWgaaWcbaGaemyAaKMaemOAaOgabeaakiabg2da9maaqababaGaemyqae0aaSbaaSqaaiabdMgaPjabdUgaRbqabaGccqWGqbaudaWgaaWcbaGaem4AaSMaemOAaOgabeaakiaaxMaacaWLjaWaaeWaaeaacqaI0aanaiaawIcacaGLPaaaaSqaaiabdUgaRbqab0GaeyyeIuoaaaa@40DE@

which guarantees reaching a local maximum in Likelihood and minimizes

‖D−M‖2=∑ij(Dij−Mij)2.     (5)
 MathType@MTEF@5@5@+=feaafiart1ev1aaatCvAUfKttLearuWrP9MDH5MBPbIqV92AaeXatLxBI9gBaebbnrfifHhDYfgasaacH8akY=wiFfYdH8Gipec8Eeeu0xXdbba9frFj0=OqFfea0dXdd9vqai=hGuQ8kuc9pgc9s8qqaq=dirpe0xb9q8qiLsFr0=vr0=vr0dc8meaabaqaciaacaGaaeqabaqabeGadaaakeaadaqbdaqaaiabdseaejabgkHiTiabd2eanbGaayzcSlaawQa7amaaCaaaleqabaGaeGOmaidaaOGaeyypa0ZaaabuaeaadaqadaqaaiabdseaenaaBaaaleaacqWGPbqAcqWGQbGAaeqaaOGaeyOeI0Iaemyta00aaSbaaSqaaiabdMgaPjabdQgaQbqabaaakiaawIcacaGLPaaadaahaaWcbeqaaiabikdaYaaakiabc6caUiaaxMaacaWLjaWaaeWaaeaacqaI1aqnaiaawIcacaGLPaaaaSqaaiabdMgaPjabdQgaQbqab0GaeyyeIuoaaaa@4A56@

In the original NMF algorithm, elements of **A **and **P **were updated at a pace determined by the difference between **M **and **D**. For a discussion of some approaches to updating **A **and **P**, see [31]. Since no uncertainty information enters the updating rules, every matrix element involved in the updating is weighted equally. In order to take advantage of uncertainty information, we minimize the *χ*^2 ^error,

x2=∑ij((Dij−Mij)σij)2     (6)
 MathType@MTEF@5@5@+=feaafiart1ev1aaatCvAUfKttLearuWrP9MDH5MBPbIqV92AaeXatLxBI9gBaebbnrfifHhDYfgasaacH8akY=wiFfYdH8Gipec8Eeeu0xXdbba9frFj0=OqFfea0dXdd9vqai=hGuQ8kuc9pgc9s8qqaq=dirpe0xb9q8qiLsFr0=vr0=vr0dc8meaabaqaciaacaGaaeqabaqabeGadaaakeaacqWG4baEdaahaaWcbeqaaiabikdaYaaakiabg2da9maaqafabaWaaeWaaeaadaWcaaqaamaabmaabaGaemiraq0aaSbaaSqaaiabdMgaPjabdQgaQbqabaGccqGHsislcqWGnbqtdaWgaaWcbaGaemyAaKMaemOAaOgabeaaaOGaayjkaiaawMcaaaqaaGGaciab=n8aZnaaBaaaleaacqWGPbqAcqWGQbGAaeqaaaaaaOGaayjkaiaawMcaaaWcbaGaemyAaKMaemOAaOgabeqdcqGHris5aOWaaWbaaSqabeaacqaIYaGmaaGccaWLjaGaaCzcamaabmaabaGaeGOnaydacaGLOaGaayzkaaaaaa@4B00@

instead of the distance between **D **and **M**. The update rules can easily incorporate this change with

Pαμ←Pαμ∑iAiαDiμσiμ∑iAiαMiμσiμ     (7)
 MathType@MTEF@5@5@+=feaafiart1ev1aaatCvAUfKttLearuWrP9MDH5MBPbIqV92AaeXatLxBI9gBaebbnrfifHhDYfgasaacH8akY=wiFfYdH8Gipec8Eeeu0xXdbba9frFj0=OqFfea0dXdd9vqai=hGuQ8kuc9pgc9s8qqaq=dirpe0xb9q8qiLsFr0=vr0=vr0dc8meaabaqaciaacaGaaeqabaqabeGadaaakeaacqWGqbaudaWgaaWcbaacciGae8xSdeMae8hVd0gabeaakiabgcziSkabdcfaqnaaBaaaleaacqWFXoqycqWF8oqBaeqaaOWaaSaaaeaadaaeqaqaaiabdgeabnaaBaaaleaacqWGPbqAcqWFXoqyaeqaaOWaaSaaaeaacqWGebardaWgaaWcbaGaemyAaKMae8hVd0gabeaaaOqaaiab=n8aZnaaBaaaleaacqWGPbqAcqWF8oqBaeqaaaaaaeaacqWGPbqAaeqaniabggHiLdaakeaadaaeqaqaaiabdgeabnaaBaaaleaacqWGPbqAcqWFXoqyaeqaaOWaaSaaaeaacqWGnbqtdaWgaaWcbaGaemyAaKMae8hVd0gabeaaaOqaaiab=n8aZnaaBaaaleaacqWGPbqAcqWF8oqBaeqaaaaaaeaacqWGPbqAaeqaniabggHiLdaaaOGaaCzcaiaaxMaadaqadaqaaiabiEda3aGaayjkaiaawMcaaaaa@5D78@

Aδα←Aδα∑jDδjσδjPαj∑jMδjσδjPαj     (8)
 MathType@MTEF@5@5@+=feaafiart1ev1aaatCvAUfKttLearuWrP9MDH5MBPbIqV92AaeXatLxBI9gBaebbnrfifHhDYfgasaacH8akY=wiFfYdH8Gipec8Eeeu0xXdbba9frFj0=OqFfea0dXdd9vqai=hGuQ8kuc9pgc9s8qqaq=dirpe0xb9q8qiLsFr0=vr0=vr0dc8meaabaqaciaacaGaaeqabaqabeGadaaakeaacqWGbbqqdaWgaaWcbaacciGae8hTdqMae8xSdegabeaakiabgcziSkabdgeabnaaBaaaleaacqWF0oazcqWFXoqyaeqaaOWaaSaaaeaadaaeqaqaamaalaaabaGaemiraq0aaSbaaSqaaiab=r7aKjabdQgaQbqabaaakeaacqWFdpWCdaWgaaWcbaGae8hTdqMaemOAaOgabeaaaaGccqWGqbaudaWgaaWcbaGae8xSdeMaemOAaOgabeaaaeaacqWGQbGAaeqaniabggHiLdaakeaadaaeqaqaamaalaaabaGaemyta00aaSbaaSqaaiab=r7aKjabdQgaQbqabaaakeaacqWFdpWCdaWgaaWcbaGae8hTdqMaemOAaOgabeaaaaGccqWGqbaudaWgaaWcbaGae8xSdeMaemOAaOgabeaaaeaacqWGQbGAaeqaniabggHiLdaaaOGaaCzcaiaaxMaadaqadaqaaiabiIda4aGaayjkaiaawMcaaaaa@5D24@

Mij=∑kAikPkj     (9)
 MathType@MTEF@5@5@+=feaafiart1ev1aaatCvAUfKttLearuWrP9MDH5MBPbIqV92AaeXatLxBI9gBaebbnrfifHhDYfgasaacH8akY=wiFfYdH8Gipec8Eeeu0xXdbba9frFj0=OqFfea0dXdd9vqai=hGuQ8kuc9pgc9s8qqaq=dirpe0xb9q8qiLsFr0=vr0=vr0dc8meaabaqaciaacaGaaeqabaqabeGadaaakeaacqWGnbqtdaWgaaWcbaGaemyAaKMaemOAaOgabeaakiabg2da9maaqababaGaemyqae0aaSbaaSqaaiabdMgaPjabdUgaRbqabaGccqWGqbaudaWgaaWcbaGaem4AaSMaemOAaOgabeaaaeaacqWGRbWAaeqaniabggHiLdGccaWLjaGaaCzcamaabmaabaGaeGyoaKdacaGLOaGaayzkaaaaaa@40DD@

where *σ*_*ij *_is the uncertainty measurement for *D*_*ij*_. This requires the existence of a new matrix, **U**, that provides estimates of the uncertainties for all data points. One advantage of this approach is that missing data is easily handled by assigning a value of 0 (for single channel) or 1.0 (for two channel, i.e. ratio) together with a large uncertainty. This essentially allows the algorithm to ignore these data points when fitting the model.

It is straightforward to follow the procedure described by Lee and Seung [30] to verify that *χ*^2 ^is nonincreasing under the modified update rules.

### Implementation of NMF and LS-NMF algorithms

The NMF algorithm was obtained from the Broad Institute as a Matlab script [15]. We converted this to a C++ version allowing modification, and we validated the C++ version by making sure we obtained the same results as for the Matlab version in a number of simulations. For LS-NMF, Equations 7–9 were implemented within C++. The code included modified update rules that take into account the additional U matrix. The code is designed for use on Beowulf clusters running Linux, and source code is available under the GNU Lesser Public License from the Fox Chase Bioinformatics web site  by following the Open-Source Software link.

Since the update rules in NMF and LS-NMF only guarantee a local minimum, the algorithms may or may not converge to the same solution on each simulation, depending on the properties of the probability distribution. To address this limitation, any simulation must be repeated multiple times (typically 20–100 individual runs) starting with different initial **A **and **P **matrices [13,15]. After the simulation, either the best [13] or average [15] factorization is selected for further analysis. We repeated NMF and LS-NMF simulations on both the cell cycle dataset and the Rosetta dataset 20 times for each dimension, *K*, between 3 and 20. Each single simulation was run for 20,000 update steps or until it converged based on a predefined *χ*^2 ^threshold (or RMSD threshold for NMF). The factorization with the lowest *χ*^2 ^error (or RMSD error for NMF) from the 20 repeated runs for each dimension was selected for further analysis. The errors were calculated as

x2=∑i=1I∑j=1J{1σij2(Dij−Mij)2}     (10)
 MathType@MTEF@5@5@+=feaafiart1ev1aaatCvAUfKttLearuWrP9MDH5MBPbIqV92AaeXatLxBI9gBaebbnrfifHhDYfgasaacH8akY=wiFfYdH8Gipec8Eeeu0xXdbba9frFj0=OqFfea0dXdd9vqai=hGuQ8kuc9pgc9s8qqaq=dirpe0xb9q8qiLsFr0=vr0=vr0dc8meaabaqaciaacaGaaeqabaqabeGadaaakeaacqWG4baEdaahaaWcbeqaaiabikdaYaaakiabg2da9maaqahabaWaaabCaeaadaGadaqaamaalaaabaGaeGymaedabaacciGae83Wdm3aa0baaSqaaiabdMgaPjabdQgaQbqaaiabikdaYaaaaaGcdaqadaqaaiabdseaenaaBaaaleaacqWGPbqAcqWGQbGAaeqaaOGaeyOeI0Iaemyta00aaSbaaSqaaiabdMgaPjabdQgaQbqabaaakiaawIcacaGLPaaadaahaaWcbeqaaiabikdaYaaaaOGaay5Eaiaaw2haaiaaxMaacaWLjaWaaeWaaeaacqaIXaqmcqaIWaamaiaawIcacaGLPaaaaSqaaiabdQgaQjabg2da9iabigdaXaqaaiabd2eanbqdcqGHris5aaWcbaGaemyAaKMaeyypa0JaeGymaedabaGaemOta4eaniabggHiLdaaaa@56FA@

RMSD=∑i=1I∑j=1J(Dij−Mij)2     (11)
 MathType@MTEF@5@5@+=feaafiart1ev1aaatCvAUfKttLearuWrP9MDH5MBPbIqV92AaeXatLxBI9gBaebbnrfifHhDYfgasaacH8akY=wiFfYdH8Gipec8Eeeu0xXdbba9frFj0=OqFfea0dXdd9vqai=hGuQ8kuc9pgc9s8qqaq=dirpe0xb9q8qiLsFr0=vr0=vr0dc8meaabaqaciaacaGaaeqabaqabeGadaaakeaacqWGsbGucqWGnbqtcqWGtbWucqWGebarcqGH9aqpdaaeWbqaamaaqahabaWaaeWaaeaacqWGebardaWgaaWcbaGaemyAaKMaemOAaOgabeaakiabgkHiTiabd2eannaaBaaaleaacqWGPbqAcqWGQbGAaeqaaaGccaGLOaGaayzkaaWaaWbaaSqabeaacqaIYaGmaaGccaWLjaGaaCzcamaabmaabaGaeGymaeJaeGymaedacaGLOaGaayzkaaaaleaacqWGQbGAcqGH9aqpcqaIXaqmaeaacqWGnbqta0GaeyyeIuoaaSqaaiabdMgaPjabg2da9iabigdaXaqaaiabd6eaobqdcqGHris5aaaa@500E@

### Data preprocessing

Two data sets were analyzed using LS-NMF and NMF, the yeast cell cycle data set [24] and the Rosetta compendium [25]. The cell cycle data set comprises measurements of mRNA levels using Affymetrix GeneChips. Synchronization was done using a temperature sensitive mutant of cdc28, which is required for passage into the late G 1 stage of the cell cycle. Cultures were grown following temperature change to activate cdc28, and mRNA was harvested at 10 minute intervals. The data was preprocessed by the original authors to identify genes that had cell cycle periodicity, resulting in a data set with 788 genes measured at 17 time points. The 10 minute intervals beginning with *t *= 0 on release from cell cycle arrest ended at 160 minutes, providing roughly two passes through the yeast cell cycle.

The Rosetta data comprises genome-wide measurements of gene expression across 300 deletion mutants or chemical treatments using oligonucleotide microarrays. We downloaded the data from Rosetta Inpharmatics, filtered it to remove experiments where less than 2 genes underwent 3-fold changes, and finally removed genes that did not change by 3-fold across the remaining conditions, resulting in 764 gene probes and 228 conditions. The Rosetta error model, based on replicates and 63 control replications of wildtype yeast, provided the estimation of uncertainty for each data point [25]. As the data comprised log-ratios, data transformation was used to convert these measurements to positive ratios and errors were propagated from the log space to the ratio space.

### Evaluating the algorithms

The most reliable criteria to evaluate algorithms applied to microarray data is the ability to recover the knowledge of biological relationships between genes, i.e., whether the suggested metagenes summarize biological knowledge. While there are few well-established benchmarks available to test the validity of metagenes, we use two well studied data sets and biological knowledge of coregulation or functional relationships to evaluate performance. For the first data set, the cell cycle data, the coregulation groups are based on biological knowledge of the yeast cell cycle and comprise 9 overlapping groups with 43 genes [28]. For the second data set, the Rosetta compendium, accuracy of the metagenes was estimated using ROC analysis based on KEGG metabolic pathways [32] and predictions of gene relationships compared to MIPS functional classification [33].

To identify membership of genes in each metagene, a threshold must be set. To do this, each row of **P **was normalized to sum to 1, and a correction factor was applied to the corresponding column of **A **to leave **M **unchanged. In order to find the metagenes in **A**, the *Z*-score was calculated for each element in each row by

Zik=Aik−μiσi,     (12)
 MathType@MTEF@5@5@+=feaafiart1ev1aaatCvAUfKttLearuWrP9MDH5MBPbIqV92AaeXatLxBI9gBaebbnrfifHhDYfgasaacH8akY=wiFfYdH8Gipec8Eeeu0xXdbba9frFj0=OqFfea0dXdd9vqai=hGuQ8kuc9pgc9s8qqaq=dirpe0xb9q8qiLsFr0=vr0=vr0dc8meaabaqaciaacaGaaeqabaqabeGadaaakeaacqWGAbGwdaWgaaWcbaGaemyAaKMaem4AaSgabeaakiabg2da9maalaaabaGaemyqae0aaSbaaSqaaiabdMgaPjabdUgaRbqabaGccqGHsisliiGacqWF8oqBdaWgaaWcbaGaemyAaKgabeaaaOqaaiab=n8aZnaaBaaaleaacqWGPbqAaeqaaaaakiabcYcaSiaaxMaacaWLjaWaaeWaaeaacqaIXaqmcqaIYaGmaiaawIcacaGLPaaaaaa@4303@

where *μ*_*i *_is the average value for gene *i *in **A**, and *σ*_*i *_is the standard deviation. The same data transformation was done also for **P**, but it was column-based to obtain behavior across metagenes for each condition. Then we assigned gene *i *as a member of metagene *k*, if *Z*_*ik*_was greater than a threshold, *T*. By changing the threshold value for the *Z*-score, we calculated an ROC curve using the procedure outlined below. It is useful to note that each gene may be assigned to multiple metagenes, which allows identification of multiple regulation of genes.

1. **Assign genes to metagenes based on the selected threshold. **Generate a Boolean connectivity matrix **C **with dimension of *I *× *K *with *C*_*ik *_= 1, iff gene *i *was assign to metagene *k*.

2. **Assign metagenes to biologically verified coregulation groups. **Metagene *k *is assigned to represent coregulation group *G*_*m *_iff the metagene maximizes ∑i∈GmCik
 MathType@MTEF@5@5@+=feaafiart1ev1aaatCvAUfKttLearuWrP9MDH5MBPbIqV92AaeXatLxBI9gBaebbnrfifHhDYfgasaacH8akY=wiFfYdH8Gipec8Eeeu0xXdbba9frFj0=OqFfea0dXdd9vqai=hGuQ8kuc9pgc9s8qqaq=dirpe0xb9q8qiLsFr0=vr0=vr0dc8meaabaqaciaacaGaaeqabaqabeGadaaakeaadaaeqbqaaiabdoeadnaaBaaaleaacqWGPbqAcqWGRbWAaeqaaaqaaiabdMgaPjabgIGiolabdEeahnaaBaaameaacqWGTbqBaeqaaaWcbeqdcqGHris5aaaa@384A@ among all possible *k*, i.e. metagene *k *is the most enriched metagene for genes in group *G*_*m*_.

3. **Keep a Boolean correlation matrix R with dimension of ***K *× *M*. The value *M *is set by the number of coregulation groups, and *R*_*km *_= 1 iff metagene *k *was assigned to represent coregulation group *G*_*m*_.

4. **Calculate the sensitivity and specificity**. The true positive, true negatives, false positives, and false negatives are given by

TP=∑k=1K∑m=1M{Rkm∑g∈GmCgm}TN=∑k=1K∑m=1M{Rkm∑g∉Gm(1−Cgm)}FP=∑k=1K∑m=1M{Rkm∑g∉GmCgm}FN=∑k=1K∑m=1M{Rkm∑g∈Gm(1−Cgm)}     (13)
 MathType@MTEF@5@5@+=feaafiart1ev1aaatCvAUfKttLearuWrP9MDH5MBPbIqV92AaeXatLxBI9gBaebbnrfifHhDYfgasaacH8akY=wiFfYdH8Gipec8Eeeu0xXdbba9frFj0=OqFfea0dXdd9vqai=hGuQ8kuc9pgc9s8qqaq=dirpe0xb9q8qiLsFr0=vr0=vr0dc8meaabaqaciaacaGaaeqabaqabeGadaaakeaafaqaaeabbaaaaeaacqWGubavcqWGqbaucqGH9aqpdaaeWbqaamaaqahabaGaei4EaSNaemOuai1aaSbaaSqaaiabdUgaRjabd2gaTbqabaGcdaaeqbqaaiabdoeadnaaBaaaleaacqWGNbWzcqWGTbqBaeqaaaqaaiabdEgaNjabgIGiolabdEeahnaaBaaameaacqWGTbqBaeqaaaWcbeqdcqGHris5aOGaeiyFa0haleaacqWGTbqBcqGH9aqpcqaIXaqmaeaacqWGnbqta0GaeyyeIuoaaSqaaiabdUgaRjabg2da9iabigdaXaqaaiabdUealbqdcqGHris5aaGcbaGaemivaqLaemOta4Kaeyypa0ZaaabCaeaadaaeWbqaaiabcUha7jabdkfasnaaBaaaleaacqWGRbWAcqWGTbqBaeqaaOWaaabuaeaadaqadaqaaiabigdaXiabgkHiTiabdoeadnaaBaaaleaacqWGNbWzcqWGTbqBaeqaaaGccaGLOaGaayzkaaaaleaacqWGNbWzcqGHjiYZcqWGhbWrdaWgaaadbaGaemyBa0gabeaaaSqab0GaeyyeIuoakiabc2ha9bWcbaGaemyBa0Maeyypa0JaeGymaedabaGaemyta0eaniabggHiLdaaleaacqWGRbWAcqGH9aqpcqaIXaqmaeaacqWGlbWsa0GaeyyeIuoaaOqaaiabdAeagjabdcfaqjabg2da9maaqahabaWaaabCaeaacqGG7bWEcqWGsbGudaWgaaWcbaGaem4AaSMaemyBa0gabeaakmaaqafabaGaem4qam0aaSbaaSqaaiabdEgaNjabd2gaTbqabaaabaGaem4zaCMaeyycI8Saem4raC0aaSbaaWqaaiabd2gaTbqabaaaleqaniabggHiLdGccqGG9bqFaSqaaiabd2gaTjabg2da9iabigdaXaqaaiabd2eanbqdcqGHris5aaWcbaGaem4AaSMaeyypa0JaeGymaedabaGaem4saSeaniabggHiLdaakeaacqWGgbGrcqWGobGtcqGH9aqpdaaeWbqaamaaqahabaGaei4EaSNaemOuai1aaSbaaSqaaiabdUgaRjabd2gaTbqabaGcdaaeqbqaamaabmaabaGaeGymaeJaeyOeI0Iaem4qam0aaSbaaSqaaiabdEgaNjabd2gaTbqabaaakiaawIcacaGLPaaaaSqaaiabdEgaNjabgIGiolabdEeahnaaBaaameaacqWGTbqBaeqaaaWcbeqdcqGHris5aOGaeiyFa0haleaacqWGTbqBcqGH9aqpcqaIXaqmaeaacqWGnbqta0GaeyyeIuoaaSqaaiabdUgaRjabg2da9iabigdaXaqaaiabdUealbqdcqGHris5aaaakiaaxMaacaWLjaWaaeWaaeaacqaIXaqmcqaIZaWmaiaawIcacaGLPaaaaaa@C6C3@

yielding sensitivity and specificity given by

Sens=TPTP+FNSpec=TNTN+FP.     (14)
 MathType@MTEF@5@5@+=feaafiart1ev1aaatCvAUfKttLearuWrP9MDH5MBPbIqV92AaeXatLxBI9gBaebbnrfifHhDYfgasaacH8akY=wiFfYdH8Gipec8Eeeu0xXdbba9frFj0=OqFfea0dXdd9vqai=hGuQ8kuc9pgc9s8qqaq=dirpe0xb9q8qiLsFr0=vr0=vr0dc8meaabaqaciaacaGaaeqabaqabeGadaaakeaafaqabeGabaaabaGaem4uamLaemyzauMaemOBa4Maem4CamNaeyypa0ZaaSaaaeaacqWGubavcqWGqbauaeaacqWGubavcqWGqbaucqGHRaWkcqWGgbGrcqWGobGtaaaabaGaem4uamLaemiCaaNaemyzauMaem4yamMaeyypa0ZaaSaaaeaacqWGubavcqWGobGtaeaacqWGubavcqWGobGtcqGHRaWkcqWGgbGrcqWGqbauaaaaaiabc6caUiaaxMaacaWLjaWaaeWaaeaacqaIXaqmcqaI0aanaiaawIcacaGLPaaaaaa@4EA8@

5. **Plot the ROC curve**. The ROC curve is generated by plotting multiple esitmates of the *Sens *and 1 – *Spec *based on different thresholds *T*. We used the trapezoidal rule to approximate the area under ROC curve.

In order to identify TN, TP, FP, FN values for Equation 13, a gold standard is required. For figures 3a and 3b, the cell cycle coregulation groups identified previously from known transcriptional response and transcription factor binding analyses in the yeast cell cycle are used [28]. For 3c, these groups are not useful, since most of these genes play a criticial role in the cell cycle and are not included in the deletion mutant set (only 5 of 43 genes are included as deletion mutants). For this data set, we instead created coreguation groups from the KEGG metabolic pathways [32]. These groups are based on the assumption that genes encoding enzymes that together provide a metabolic pathway are likely to be coexpressed. The 11 groups comprising 63 genes are provided in Table 1.

The second way that we evaluated the performance of NMF and LS-NMF was by predicting gene relationships as done by Kim and Tidor [13]. We assumed that similarity of gene expression profiles as measured by metagenes for different deletion mutants indicated a functional relationship between the deleted genes. We calculated predictions in both the original, unreduced data space (from the **D **matrix) and in the reduced dimensional spaces computed by NMF, LS-NMF, and NMF applied to scaled data (from the **P **matrix). The scaled data was produced by taking the Rosetta data and dividing by the uncertainty estimates. The Pearson correlation coefficient was calculated between all conditions (i.e. deletion mutants), and the absolute value of the correlation coefficient was used as a score for the predicted relationship. The functional relationships between deletion mutants are used as predictors of functional relationships for the deleted genes. From the Rosetta dataset, Pearson correlation coefficients were calculated for all possible pairs of 215 columns in **P **(i.e, all possible deletion mutant combinations). The scores were compared to a threshold for multiple thresholds, and successful predictions of functional relationships were determined by comparison to the MIPS Funcat annotation [33,34]. The Funcat annotations are hierarchical, comprising high level (level 1, example 41 DEVELOPMENT) to low level (level 4, example 41.01.03.03 mycelium development) categorizations. In order to have sufficient data for the analysis and to have sufficient fine resolution of function, we focused on the second and third levels of annotation (e.g., 41.01 fungal/microorganismic development, 41.01.03 tissue pattern formation). Two genes appearing in the same MIPS functional category were considered as functionally related. For all combinations of two genes predicted as functionally related by the Pearson correlation test of their deletion mutants, the total number of successful predictions were determined. The fraction of successful predictions were then plotted as the threshold was reduced and the number of predictions increased. In cases where genes do not have a MIPS functional assignment at the desired level or were classified as "UNCLASSIFIED PROTEINS", the corresponding deletion mutants were removed from the analysis. This left 168 genes at level 3, where MIPS has 441 different classifications, and 180 genes at level 2, where MIPS has 158 different classifications.

## Authors' contributions

GW performed all analyses presented in this work, developed the new update equations for LS-NMF, and coded the LS-NMF algorithm. AVK analyzed the KEGG metabolic pathways and created the genes lists presented in Table 1 by comparison to the Rosetta deletion mutant data. MFO oversaw the project and suggested the use of the *χ*^2 ^measure to improve NMF.

## Figures and Tables

**Table 1:** Coregulation Groups for Rosetta ROC Analysis. This table provides groups of genes that are believed to be coregulated based on the metabolic pathways of *S. cerevisiae *as summarized in the KEGG database.

Coregulation Groups		
Group	KEGG Pathway	Genes

1	glucose fermentation	ADH5 ALD5 ADH4 ADH2 ADH1 ALD4
2	phenylalanine degradation	ADH5 ARO10 ADH4 ARO9 ADH2 ADH1
3	sulfate assimilation pathway II	MET10 MET3 ECM17 MET14 MET17 MET16
4	gluconeogenesis	TDH2 PCK1 YMR323W ERR1 ERR2
5	serine-isocitrate lyase pathway	CIT2 ACO1 YMR323W ERR1 ERR2
6	TCA cycle, aerobic respiration	CIT2 KGD2 ACO1 IDH1 IDH2
7	tryptophan degradation	ADH5 ARO10 ADH4 ADH2 ADH1
8	glycolysis	TDH2 YMR323W ERR1 ERR2
9	histidine biosynthesis	HIS7 HIS4 HIS5 HIS3
10	leucine biosynthesis	LEU2 LEU1 BAT1 LEU4
11	tryptophan degradation via kynurenine	BNA4 BNA1 BNA2 BNA5
